# A large-scale dataset of choice and response-time data in intertemporal choice

**DOI:** 10.1038/s41597-026-06947-4

**Published:** 2026-03-03

**Authors:** Hannah Pongratz, Martin Schoemann

**Affiliations:** 1 Department of Psychology, University of Mannheim, 68131 Mannheim, Germany; 2 Department of Psychology, TUD Dresden University of Technology, 01062 Dresden, Germany

**Keywords:** Human behaviour, Databases

## Abstract

The study of intertemporal choices (ITC) plays a vital role in psychological and behavioral economics research. Models of intertemporal choice (ITC) have traditionally focused on choices. A growing interest in the underlying cognitive processes has initiated the development of process models. Process models require process data, and yet ITC research has largely overlooked even the simplest process data – response times (RTs). We present a large-scale dataset of choices and response times from 100 ITC studies with 11,852 subjects and 1,172,644 trials. In addition to behavioral data, we collected various methodological and sample information (e.g., task procedure, incentivization). The objective of the large-scale dataset is to facilitate the development of more nuanced and accurate theories of ITC. The associated ITC Database is open to ongoing submissions and is projected to expand continuously.

## Background & Summary

Intertemporal choice (*ITC*) and the corresponding phenomenon of temporal (or delay) discounting have been extensively studied across disciplines, with roots in economics and subsequent exploration in psychology. Initially, economic models of ITC focused on normative or descriptive accounts of ITC behavior from a discounted utility perspective^1–3^, neglecting the underlying psychological processes that drive temporal discounting^4–6^. In contrast, psychological investigations have sought to explain the cognitive and emotional mechanisms that influence ITC^7–10^, often incorporating process-level assumptions or mechanisms into their models, such as alternative-wise vs. attribute-wise information processing^11–13^ or evidence accumulation^14–17^. However, these psychological models have rarely moved beyond choice data, despite the need for process-level data to inform and validate assumptions and mechanisms in process models^18,19^. Response time (*RT*) data, as a basic form of process data^20^, can complement choice data, putting important constraints on process models and hence providing valuable insights into the psychological processes underlying ITC^21^.

The development of good process models requires the identification of robust effects on process data, and such effects are commonly robust to heterogeneity in the data^22,23^. Heterogeneity, in this context, refers to the variation in research results that exceeds expected sampling error, indicating a lack of coherence between applied concepts and observed data, which reflects an incomplete understanding of the phenomenon in particular^24,25^ and a generalizability crisis in general^26^. To address this challenge, researchers can employ meta-studies, a recent research approach that involves creating a set of many small studies (micro-studies), each with a comparatively small number of participants or observations, all based on the same fundamental design but varying in their details to systematically replicate the same basic experiment^27–29^. This approach allows researchers to assess the phenomenon of interest while also examining the causal effects of moderators and ensuring generalizability^23,26^.

While meta-studies offer an efficient and effective way to achieve the benefits of traditional replication and meta-analysis, they are typically conducted prospectively, requiring new data collection and incurring additional costs. However, meta-studies can also be conducted retrospectively, leveraging existing, yet unevaluated data, thereby eliminating the need for new data collection and associated costs. This retrospective approach is particularly well-suited for traditional basic experiments, such as those used to study ITC and delay discounting, where a large amount of data is already available but certain aspects or variables have remained unused. In such cases, retrospective meta-studies may be considered economically and ethically mandatory, as they can provide a comprehensive understanding of the phenomenon without incurring additional costs or burdening participants. Importantly, retrospective meta-studies can mitigate publication bias, as they may utilize aspects of the data from peer-reviewed publications that have not been previously evaluated, thereby providing a valid and representative picture of the research landscape. By harnessing the power of existing data, retrospective meta-studies can cover a significant proportion of the heterogeneity in ITC research, making them an attractive approach for researchers seeking to advance our understanding of this complex phenomenon.

To address this need, we have compiled a large-scale dataset of choices and response times in standard ITC tasks—the *ITC Database*—drawn from a diverse sample that covers a significant proportion of the methodological variation in ITC research. By making this large-scale dataset available, we aim to facilitate the advancement of ITC research by promoting the development of more nuanced and accurate theories of ITC. The large-scale dataset described here offers an opportunity for researchers to explore the complexities of ITC behavior, examine the robustness of effects across different experimental designs and participant populations, and develop more comprehensive process models that can account for the heterogeneity in ITC and temporal discounting.

The dataset has not been used in any previous publication. However, the Confidence Database^30^ is a similar endeavor, representing a comprehensive repository designed to facilitate the study of confidence across various cognitive and perceptual domains, for instance, to explore the relationship between confidence and accuracy in decision-making processes^31^. Another related endeavor, is the recent Attentional Control Data Collection^32^, representing a structured collections of open data to allow a larger community of researchers easy access to a large body of data from attentional control experiments. Our large-scale dataset, the Confidence Database^30^, and the Attentional Control Data Collection^32^ represent datasets that can be considered similar to what other large-scale endeavors, such as the Psychological Science Accelerator^33^ or the Human Connectome Project^34^, eventually produce – a highly representative and generalizable dataset of a set of phenomena of interest.

## Methods

Our large-scale dataset of choices and response times in standard ITC tasks is a secondary dataset, compiled from existing publications that have investigated ITC behavior. To identify eligible datasets, we employed a three-step procedure. First, we conducted a systematic search for publications that may be associated with eligible datasets, aiming to exhaustively cover the relevant peer-reviewed literature. Next, we collected the data by identifying and accessing associated open data repositories or by contacting authors with formal data requests. Finally, we processed the collected data by structuring each dataset in a common format that can be easily imported and analyzed in multiple software packages. As part of this processing step, we also compiled relevant metadata for each dataset, providing essential context for subsequent analyses and ensuring the transparency and reproducibility of our dataset.

### Systematic search

**Fig. 1:**
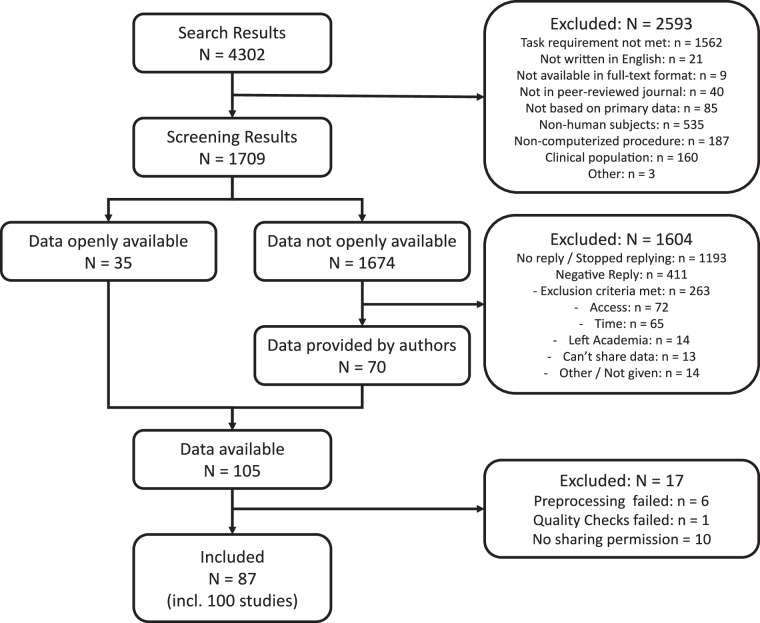
Systematic search and data collection process. Please note that multiple exclusion criteria may have applied to the same publication.

To identify eligible datasets, we followed a prespecified protocol (see https://osf.io/hsfuz) and conducted a systematic literature search (see Fig. 1), aiming to include any study that employed a standard ITC task and for which trial-level choice and response-time data may be obtained. We defined a standard ITC task as a two-alternative forced choice task with well-specified (i.e., non-probabilistic or ambiguous) monetary rewards and time delays stated as intervals. To ensure a reproducible and unbiased search, we restricted our search to two widely accepted electronic databases: PubMed and the Web of Science Core Collection. Here we deviated from our prespecified protocol as we had registered to search three databases—Scopus, PubMed, and the Web of Science Core Collection—but due to a mistake during the search we had exported only the first ten search results from Scopus which were all found illegible or being duplicates. We used a comprehensive search string that included the concepts of intertemporal choice, delay discounting, temporal discounting, and intertemporal decisions, applying appropriate wild cards and adapting the search string to each database (see Table 1). When possible, the search command included a filter for human subjects and original articles. This initial search conducted on 2024/29/02 yielded 4,302 results (excluding duplicates).

**Table 1:** Search commands used in each of the databases.

Database	Search string/command line
PubMed	(“delay discount*” [Title/Abstract] OR “temporal discount*” [Title/Abstract] OR “intertemporal choice*” [Title/Abstract] OR “intertemporal decision*” [Title/Abstract]) AND “human*” [MeSH Terms]
Web of Science	TS = (“delay discount*” OR “temporal discount*” OR “intertemporal choice*” OR “intertemporal de-cision*”) AND DT = (Article)

We then applied a series of prespecified exclusion criteria to filter out studies that did not meet our eligibility criteria. We excluded studies that did not meet our definition of an intertemporal choice task, were not written in English, were not available in full-text format, were not published in a peer-reviewed journal, or whose analyses were not based on primary data. Additionally, we excluded studies conducted in non-human subjects, those that used a non-computerized procedure (which would preclude the collection of response-time data), and those conducted solely in populations with neurological abnormalities or psychiatric disorders (although we did include control groups from such studies if they were included alongside a clinical group). After applying these exclusion criteria, we excluded 1,682 studies at the abstract screening stage and 911 studies at the full-text stage, leaving 1,709 candidate publications that met our eligibility criteria and proceeded to the next stage of data collection.

### Data collection & data processing

To collect the eligible datasets, we employed a two-pronged approach. First, we identified and accessed associated open data repositories that provided all data of interest for 35 publications, extracting and processing the data of interest (as described in the Data Record section) with the aid of the accompanying publication and any publicly available information for understanding the published data. Second, we contacted the corresponding author(s) of the remaining publications with formal data requests, sending out a total of 1,674 requests. Initially, we asked authors to submit the data of interest in a specific format, extracted and processed according to our requirements. However, to maximize data retrieval, we also accepted unprocessed data from authors, provided they supplied the necessary information to enable us to understand and work with the data. In such cases, we extracted and processed the data of interest ourselves.

Overall, we collected and processed 112 datasets from 98 publications. In a last step, we contacted all corresponding authors asking for explicit permission to publish their dataset(s) within this large-scale dataset under a CC BY-NC-SA license. In 87 cases the authors agreed to this (i.e., without any special collaboration agreements), and hence our large-scale dataset features 100 datasets from 87 publications (for references, see the Data Input section).

### Input data

**Fig. 2:**
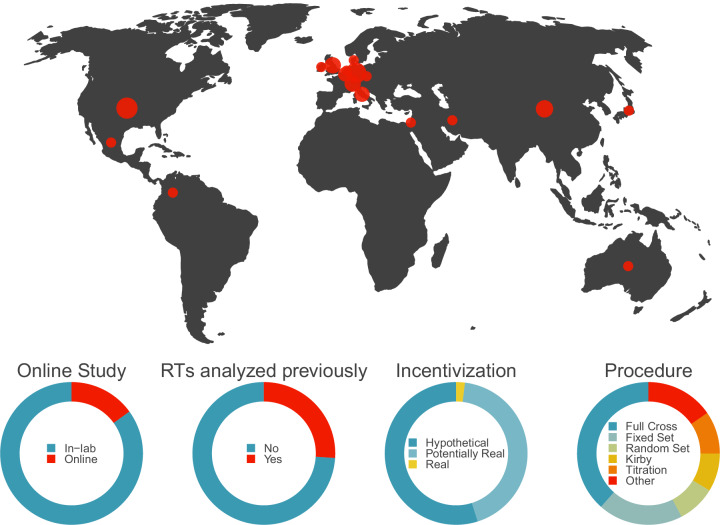
Overview of individual datasets in the large-scale dataset. Worldmap (top) shows the geographical origin of the datasets. Donut charts (bottom) show the proportion of selected study characteristics.

All literature sources for the large-scale dataset are listed in the data file and cited in this paper^8,35–112^(for an overview, see Fig. 2). Additionally, all repository sources with formal metadata (i.e., DOI) are listed in the data file and cited in this paper^113–120^. All repository sources without formal metadata are listed in the data file (i.e., hyperlinks are provided there).

## Data Record

The large-scale dataset^121^ is hosted on the Open Science Framework (OSF; https://osf.io/3wsae/) under a CC BY-NC-SA license as data file both in .csv-format and .RData-format.

The data file contains the following fields:

- ***paper***: The dataset key
- ***subj_idx***: The subject index or identifier
- ***subj_ident***: A unique identifier of a subject across the complete dataset. Use this for running subject-level analyses instead of the subj_idx, as there may be duplicated subj_idx in different studies.
- ***session***: A session index or identifier for the session in which the data was collected. In studies with a single session, this is always 1
- ***sess_ident***: A unique identifier of a session across the complete dataset
- ***trial_idx***: The trial index, indicating the order in which trials were presented (if available, otherwise = NA)
- ***ss_value***: The reward value of the smaller, sooner option
- ***ss_time***: The delay of the smaller, sooner option (in days)
- ***ll_value***: The reward value of the larger, later option
- ***ll_time***: The reward value of the larger, later option (in days)
- ***choice***: The chosen option (SS chosen = 0, LL chosen = 1)
- ***rt***: The response time of the decision in seconds
- ***subj_excl***: Indicators of whether a subject should be excluded based on our exclusion criteria (do not exclude subject = FALSE, do exclude subject = TRUE)
- ***subj_excl_ind***: The reason for exclusion of a subject (if excluded, otherwise = NA). Number(s) correspond to those in ***subject_excl_criteria***
- ***trial_excl***: Indicators of whether a trial should be excluded based on our exclusion criteria (do not exclude trial = FALSE, do exclude trial = TRUE)
- ***trial_excl_ind***: The reason for exclusion of a trial (if excluded, otherwise = NA). Number(s) correspond to those in ***trial_excl_criteria***
- ***age***: The age of the subject (if available, otherwise = NA)
- ***additional_conditions***: Any special conditions or characteristics of the subjects that are not grounds for exclusion (if present, otherwise = NA)
- ***subset***: A variable used to map data to specific entries in the dataset (i.e., in cases where parts of the dataset differ in key experimental variables)
- ***subset_label***: A short description of which parts of the data belong to this subset
- ***doi_publication***: The DOI of the respective article
- ***link_data***: The link of published data (if available, otherwise = NA)
- ***doi_data***: The DOI of the published data (if available, otherwise = NA)
- ***country***: The country in which the experiment was conducted or from which the participants were recruited in the case of online studies
- ***currency***: The currency in which the monetary reward values were presented
- ***time_unit***: The unit of time in which the delays are stored in the original data files. This is not necessarily the unit of time in which the information was presented to subjects
- ***procedure***: The method of the trial construction
- ***incentivization***: The method of choice incentivization
- ***presentation_of_information***: The method of attribute presentation in the trial
- ***additional_methods***: Any special interventions used in the study that are not grounds for exclusion (if present, otherwise = NA)
- ***additional_interventions***: Any special interventions used in the study that are not grounds for exclusion (if present, otherwise = NA)
- ***fixed_attributes***: Attribute(s) fixed for the duration of the entire study
- ***time_pressure***: Whether there was a response deadline (Yes = 1, No = 0)
- ***time_pressure_cont***: If present, how long was the response deadline (in seconds)
- ***subj_excl_criteria***: Exclusion criteria applied to subjects
- ***trial_excl_criteria***: Exclusion criteria applied to trials
- ***missing_trials_absolute***: The number of trials containing missing values on key variables (stimulus information, choice or RTs) including trials marked as excluded
- ***missing_trials_relative***: The percentage of trials containing missing values on key variables (stimulus information, choice or RTs) including trials marked as excluded
- ***online_study***: Whether the study was conducted online (Yes = 1, No = 0)
- ***rt_in_full_seconds***: Whether the response times were recorded in full seconds (Yes = 1, No = 0)
- ***rt_data_analyzed***: Whether response time data have been analyzed in the original publication (Yes = 1, No = 0)
- ***comments***: Any comments about the study

## Technical Validation

To ensure the technical quality of the individual datasets in our large-scale dataset, we performed several checks and analyses with regard to subject and trial numbers, missing values, stimulus information, choice, and response times.

We compared the stated number of subjects and trials in the publications to the actual numbers present in the datasets. We identified deviations in the number of subjects and in the number of trials in 17 and 12 datasets, respectively. We logged these in the comments. We checked the number of missing values in all fields and, where possible, compared them to the information in the publication. We identified no deviations for this. We also checked the number of trials missing information on key variables (stimulus information, choice or RTs). The average amount of such trials across all datasets is 0.45%. 65 out of the 100 studies do not have any missing values and only 12 studies are missing more than 1% of all trials.

We visually inspected the distribution of the stimulus information (i.e., *ss_value* *<* *ll_value*; *ss_time* *<* *ll_time*) to identify values that were obviously outside of the expected pattern. In these cases, we contacted the authors to verify or correct the data. In one case the discrepancy could not be resolved, and the trials in question where excluded following the authors recommendation.

**Fig. 3:**
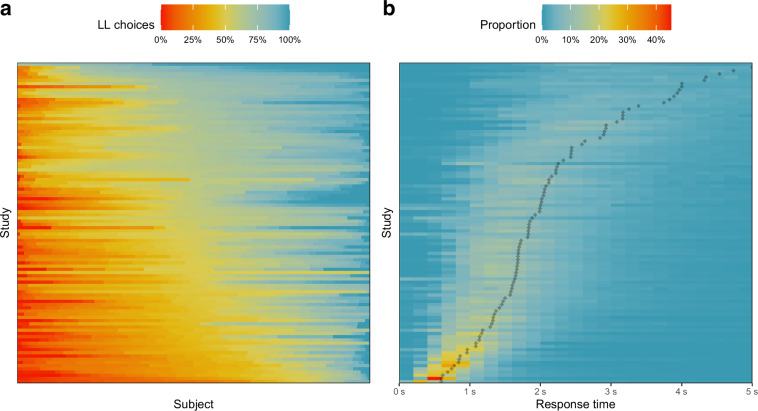
Choice and RT distribution per dataset. (**a**) Proportion of LL choices for each subject with datasets ordered by the overall proportion of LL choices. (**b**) Proportion of RTs for each RT bin across all subjects with datasets ordered by the median RT (indicated by the black diamonds).

To check the plausibility of the choice coding, we performed logistic regression analyses. Specifically, we regressed the dichotomous choice on the stimulus information and checked if the probability of choosing the larger, later option increased with increasing *ll_value*, decreasing *ss_value*, decreasing *ll_time*, and increasing *ss_time*. If half or more of the predictors did not show the expected effect, we visually inspected the choice distribution across as well as for individual subjects. If this did not resolve the issue, we contacted the authors. In one case, the discrepancy could not be resolved, and the study in question was excluded (see Fig. 3a).

To check the plausibility of the RTs, we checked for implausibly fast or slow response times (*M*_*rt*_ > 10, no negative RTs, more than 10% of RTs below 200 ms, more than 10% of RTs above 20 s if no time pressure was applied, and no RT exceeding the respective response deadline, if time pressure was applied). Most often this was due to the RTs being indicated in milliseconds (ms) instead of seconds (s). However, in two instances, this was not the case. For these, we contacted the authors to resolve the issue, which we were able to do in all cases. As the focus of this large-scale lies on RT data, we additionally evaluated the RT distributions per subject based on skewness and bi-modality index and we expected right-skewed, uni-modal RT distributions in more than 80% of the subjects. We identified no study violating this expectation (see Fig. 3b).

## Usage Notes

To enhance the long-term sustainability and impact of the large-scale dataset presented in this article, it is provided in two versions: a static version^121^ which corresponds to this article, and a continuously evolving version published as the *ITC Database*^122^. The ITC Database is open to contributions of individual datasets and will be updated indefinitely. In addition to the large-scale dataset compiled from individual datasets, the ITC Database also provides all individual datasets as separate primary data files. The code used to process the individual raw datasets is not provided because (a) some datasets may have been received in already processed form, such that the corresponding code is unavailable, and (b) permission to publish the raw datasets or processing code may not have been granted.

## Data Availability

The large-scale dataset is available at the OSF^121^ (https://osf.io/3wsae/) both in .RData- and .csv-format and can be used under a CC BY-NC-SA license.

## Code availability

The code to reproduce the tables, figures, and quality checks in this article is available at the OSF (https://osf.io/3wsae/).

## References

[1] Samuelson, P. A. A Note on Measurement of Utility. *The Review of Economic Studies* **4**, 155. 10.2307/2967612 (1937).

[2] Laibson, D. Golden Eggs and Hyperbolic Discounting. *The Quarterly Journal of Economics* **112**, 443–478. 10.1162/003355397555253 (May 1997).

[3] Loewenstein, G. & Prelec, D. Anomalies in Intertemporal Choice: Evidence and an Interpretation. *The Quarterly Journal of Economics* **107**, 573–597, 10.2307/2118482 (1992).

[4] Berns, G. S., Laibson, D. & Loewenstein, G. Intertemporal choice – toward an integrative framework. *Trends in Cognitive Sciences* **11**, 482–488, 10.1016/j.tics.2007.08.011 (2007). 17980645 10.1016/j.tics.2007.08.011

[5] Van den Bos, W. & McClure, S. M. Towards a general model of temporal discounting. *Journal of the Experimental Analysis of Behavior* **99**, 58–73, 10.1002/jeab.6 (2012). 23344988 10.1002/jeab.6 PMC8127626

[6] Loewenstein, G. & Carbone, E. Self-control = temporal discounting. *Current Opinion in Psychology* **60**, 101924, 10.1016/j.copsyc.2024.101924 (2024). 39447340 10.1016/j.copsyc.2024.101924

[7] Rick, S. & Loewenstein, G. Intangibility in intertemporal choice. *Philosophical Transactions of the Royal Society B: Biological Sciences* **363**, 3813–3824, 10.1098/rstb.2008.0150 (2008) 10.1098/rstb.2008.0150 PMC2607360 18829432

[8] Lempert, K. M. & Phelps, E. A. The Malleability of Intertemporal Choice. *Trends in Cognitive Sciences* **20**, 64–74, 10.1016/j.tics.2015.09.005 (2016). 26483153 10.1016/j.tics.2015.09.005 PMC4698025

[9] Zauberman, G. & Urminsky, O. Consumer intertemporal preferences. *Current Opinion in Psychology* **10**, 136–141, 10.1016/j.copsyc.2016.01.005 (2016).

[10] Bulley, A. & Schacter, D. L. Deliberating trade-offs with the future. *Nature Human Behaviour* **4**, 238–247, 10.1038/s41562-020-0834-9 (2020). 32184495 10.1038/s41562-020-0834-9 PMC7147875

[11] González-Vallejo, C. Making trade-offs: A probabilistic and context-sensitive model of choice behavior. *Psychological Review* **109**, 137–155, 10.1037/0033-295X.109.1.137 (2002). 11863035 10.1037/0033-295x.109.1.137

[12] Scholten, M. & Read, D. The psychology of intertemporal tradeoffs. *Psychological Review* **117**, 925–944, 10.1037/a0019619 (2010). 20658858 10.1037/a0019619

[13] Amasino, D. R., Sullivan, N. J., Kranton, R. E. & Huettel, S. A. Amount and time exert independent influences on intertemporal choice. *Nature Human Behaviour* **3**, 383–392, 10.1038/s41562-019-0537-2 (2019). 30971787 10.1038/s41562-019-0537-2 PMC8020819

[14] Dai, J. & Busemeyer, J. R. A probabilistic, dynamic, and attribute-wise model of intertemporal choice. *Journal of Experimental Psychology: General* **143**, 1489–1514, 10.1037/a0035976 (2014). 24635188 10.1037/a0035976 PMC4115005

[15] Dai, J., Pleskac, T. J. & Pachur, T. Dynamic cognitive models of intertemporal choice. *Cognitive Psychology* **104**, 29–56, 10.1016/j.cogpsych.2018.03.001 (2018). 29587183 10.1016/j.cogpsych.2018.03.001

[16] Rodriguez, C. A., Turner, B. M. & McClure, S. M. Intertemporal Choice as Discounted Value Accumulation. *PLoS ONE* **9**, e90138, 10.1371/journal.pone.0090138 (2014). 24587243 10.1371/journal.pone.0090138 PMC3938649

[17] He, L., Wall, D., Reeck, C. & Bhatia, S. Information acquisition and decision strategies in intertemporal choice. *Cognitive Psychology* **142**, 101562, 10.1016/j.cogpsych.2023.101562 (2023). 36996641 10.1016/j.cogpsych.2023.101562

[18] Schulte-Mecklenbeck, M., Kühberger, A. & Ranyard, R. The role of process data in the development and testing of process models of judgment and decision making. *Judgment and Decision Making* **6**, 733–739, 10.1017/s1930297500004162 (2011).

[19] Johnson, E. J., Schulte-Mecklenbeck, M. & Willemsen, M. C. Process models deserve process data: Comment on Brandstätter, Gigerenzer, and Hertwig (2006). *Psychological Review* **115**, 263–272, 10.1037/0033-295x.115.1.263 (2008). 18211202 10.1037/0033-295X.115.1.263

[20] Luce, R. D. *Response times* (Oxford University Press, New York, 1986).

[21] Donkin, C. & Brown, S. D. in *Stevens’ Handbook of Experimental Psychology and Cognitive Neuroscience* (ed Wixted, J. T.) 1–33. 10.1002/9781119170174.epcn509 (Wiley, Mar. 2018).

[22] Bryan, C. J., Tipton, E. & Yeager, D. S. Behavioural science is unlikely to change the world without a heterogeneity revolution. *Nature Human Behaviour* **5**, 980–989, 10.1038/s41562-021-01143-3 (2021). 34294901 10.1038/s41562-021-01143-3 PMC8928154

[23] Bolger, N., Zee, K. S., Rossignac-Milon, M. & Hassin, R. R. Causal processes in psychology are heterogeneous. *Journal of Experimental Psychology: General* **148**, 601–618, 10.1037/xge0000558 (2019). 30973259 10.1037/xge0000558

[24] Linden, A. H. & Hönekopp, J. Heterogeneity of Research Results: A New Perspective From Which to Assess and Promote Progress in Psychological Science. *Perspectives on Psychological Science* **16**, 358–376, 10.1177/1745691620964193 (2021). 33400613 10.1177/1745691620964193 PMC7961629

[25] Nunes, A., Trappenberg, T. & Alda, M. The definition and measurement of heterogeneity. *Translational Psychiatry* **10**. 10.1038/s41398-020-00986-0 (Aug. 2020). 10.1038/s41398-020-00986-0 PMC7445182 32839448

[26] Yarkoni, T. The generalizability crisis. *Behavioral and Brain Sciences* **45**. ISSN: 1469-1825. 10.1017/S0140525X20001685 (2020). 10.1017/S0140525X20001685 PMC10681374 33342451

[27] Baribault, B. *et al*. Metastudies for robust tests of theory. *Proceedings of the National Academy of Sciences* **115**, 2607–2612. 10.1073/pnas.1708285114 (Mar. 2018). 10.1073/pnas.1708285114 PMC5856505 29531092

[28] De Boeck, P. & Jeon, M. Perceived crisis and reforms: Issues, explanations, and remedies. *Psychological Bulletin* **144**, 757–777. 10.1037/bul0000154 (July 2018). 10.1037/bul0000154 29771554

[29] DeKay, M. L., Rubinchik, N., Li, Z. & De Boeck, P. Accelerating Psychological Science With Metastudies: A Demonstration Using the Risky-Choice Framing Effect. *Perspectives on Psychological Science* **17**, 1704–1736, 10.1177/17456916221079611 (2022). 35834353 10.1177/17456916221079611

[30] Rahnev, D. *et al*. The Confidence Database. *Nature Human Behaviour* **4**, 317–325. ISSN: 2397-3374. 10.1038/s41562-019-0813-1 (2020). 10.1038/s41562-019-0813-1 PMC7565481 32015487

[31] Jin, S., Verhaeghen, P. & Rahnev, D. Across-subject correlation between confidence and accuracy: A meta-analysis of the Confidence Database. *Psychonomic Bulletin & Review* **29**, 1405–1413, 10.3758/s13423-022-02063-7 (2022). 35129781 10.3758/s13423-022-02063-7 PMC10777204

[32] Haaf, J. M., Hoffstadt, M. & Lesche, S. Attentional control data collection: A resource for efficient data reuse. *Behavior Research Methods* **57**. ISSN: 1554-3528. 10.3758/s13428-025-02717-z (2025). 10.3758/s13428-025-02717-z PMC12187800 40555897

[33] Moshontz, H. *et al*. The Psychological Science Accelerator: Advancing Psychology Through a Distributed Collaborative Network. *Advances in Methods and Practices in Psychological Science* **1**, 501–515, 10.1177/2515245918797607 (2018). 31886452 10.1177/2515245918797607 PMC6934079

[34] Van Essen, D. *et al*. The Human Connectome Project: A data acquisition perspective. *NeuroImage* **62**, 2222–2231, 10.1016/j.neuroimage.2012.02.018 (2012). 22366334 10.1016/j.neuroimage.2012.02.018 PMC3606888

[35] Alonso-Díaz, S. *et al*. Measuring perceptions of postconflict actors’ economic behavior: The case of Colombia. *Peace and Conflict: Journal of Peace Psychology* **28**, 44–48. ISSN: 1078-1919. 10.1037/pac0000543 (2022).

[36] Alvarez, E. E., Hafezi, S., Bonagura, D., Kleiman, E. M. & Konova, A. B. A proof-of-concept ecological momentary assessment study of day-level dynamics in value-based decision-making in opioid addiction. *Frontiers in Psychiatry* **13**, 817979, 10.3389/fpsyt.2022.817979 (2022). 35664484 10.3389/fpsyt.2022.817979 PMC9156899

[37] Andersen, S., Harrison, G. W., Lau, M. I. & Rutström, E. E. Discounting behavior: A reconsideration. *European Economic Review* **71**, 15–33, 10.1016/j.euroecorev.2014.06.009 (2014).

[38] Armstrong, C. H. & Hoge, E. A. Associations of delay discounting rate with anxiety disorder symptomatology and diagnoses. *The Psychological Record* **74**, 59–74, 10.1007/s40732-023-00582-w (2024).

[39] Bahrami, R. & Borhani, K. Excluded and myopic: Social exclusion increases temporal discounting. *PloS one* **18**, e0290175, 10.1371/journal.pone.0290175 (2023). 37582119 10.1371/journal.pone.0290175 PMC10426998

[40] Becker, D., Vliek, M. L. & Sassenberg, K. Flexible control: Conflict mindsets reduce the association between trait measures of self-control decision-making and delay discounting. *Motivation Science* **9**, 21, 10.1037/mot0000283 (2023).

[41] Berenson, K. R. *et al*. Impulsivity, rejection sensitivity, and reactions to stressors in borderline personality disorder. *Cognitive Therapy and Research* **40**, 510–521, 10.1007/s10608-015-9752-y (2016). 27616800 10.1007/s10608-015-9752-y PMC5015893

[42] Bixter, M. T. & Luhmann, C. C. Evidence for implicit risk: Delay facilitates the processing of uncertainty. *Journal of Behavioral Decision Making* **28**, 347–359, 10.1002/bdm.1853 (2015).

[43] Bixter, M. T., Trimber, E. M. & Luhmann, C. C. Are intertemporal preferences contagious? Evidence from collaborative decision making. *Memory & cognition* **45**, 837–851, 10.3758/s13421-017-0698-z (2017). 28265901 10.3758/s13421-017-0698-z

[44] Bixter, M. T. & Rogers, W. A. Age-related differences in delay discounting: Immediate reward, reward magnitude, and social influence. *Journal of Behavioral Decision Making* **32**, 471–484, 10.1002/bdm.2124 (2019).

[45] Bruder, L. R., Scharer, L. & Peters, J. Reliability assessment of temporal discounting measures in virtual reality environments. *Scientific reports* **11**, 7015, 10.1038/s41598-021-86388-8 (2021). 33782424 10.1038/s41598-021-86388-8 PMC8007609

[46] Bulley, A., Lempert, K. M., Conwell, C., Irish, M. & Schacter, D. L. Intertemporal choice reflects value comparison rather than self-control: insights from confidence judgements. *Philosophical Transactions of the Royal Society B: Biological Sciences* **377**. 10.1098/rstb.2021.0338 (2022). 10.1098/rstb.2021.0338 PMC9619231 36314145

[47] Calluso, C., Tosoni, A., Cannito, L. & Committeri, G. Concreteness and emotional valence of episodic future thinking (EFT) independently affect the dynamics of intertemporal decisions. *PloS one* **14**, e0217224, 10.1371/journal.pone.0217224 (2019). 31136620 10.1371/journal.pone.0217224 PMC6538244

[48] Cao, Q., Hofmeyr, A., Hsu, E., Luo, S. & Monterosso, J. Fixed attributes and discounting behavior: Effects of holding one attribute constant during an intertemporal choice task. *Experimental Psychology* **68**, 305, 10.1027/1618-3169/a000535 (2021). 35258360 10.1027/1618-3169/a000535

[49] Castrellon, J. J. *et al*. Mesolimbic dopamine D2 receptors and neural representations of subjective value. *Scientific reports* **9**, 20229, 10.1038/s41598-019-56858-1 (2019). 31882947 10.1038/s41598-019-56858-1 PMC6934551

[50] Chiong, W. *et al*. Neuroeconomic dissociation of semantic dementia and behavioural variant frontotemporal dementia. *Brain* **139**, 578–587, 10.1093/brain/awv344 (2016). 26667277 10.1093/brain/awv344 PMC4861653

[51] Civai, C., Hawes, D. R., DeYoung, C. G. & Rustichini, A. Intelligence and extraversion in the neural evaluation of delayed rewards. *Journal of Research in Personality* **61**, 99–108, 10.1016/j.jrp.2016.02.006 (2016).

[52] Croote, D. E. *et al*. Delay discounting decisions are linked to temporal distance representations of world events across cultures. *Scientific Reports* **10**, 12913, 10.1038/s41598-020-69700-w (2020). 32737357 10.1038/s41598-020-69700-w PMC7395128

[53] Daood, M. *et al*. Fronto-striatal connectivity patterns account for the impact of methylphenidate on choice impulsivity among healthy adults. *Neuropharmacology* **216**, 109190, 10.1016/j.neuropharm.2022.109190 (2022). 35835210 10.1016/j.neuropharm.2022.109190

[54] Demurie, E., Roeyers, H., Baeyens, D. & Sonuga-Barke, E. Temporal discounting of monetary rewards in children and adolescents with ADHD and autism spectrum disorders. *Developmental science* **15**, 791–800, 10.1111/j.1467-7687.2012.01178.x (2012). 23106733 10.1111/j.1467-7687.2012.01178.x

[55] Demurie, E., Roeyers, H., Baeyens, D. & Sonuga-Barke, E. Domain-general and domain-specific aspects of temporal discounting in children with ADHD and autism spectrum disorders (ASD): A proof of concept study. *Research in developmental disabilities* **34**, 1870–1880, 10.1016/j.ridd.2013.03.011 (2013). 23578902 10.1016/j.ridd.2013.03.011

[56] Demurie, E., Roeyers, H., Wiersema, J. R. & Sonuga-Barke, E. No evidence for inhibitory deficits or altered reward processing in ADHD: data from a new integrated monetary incentive delay go/no-go task. *Journal of attention disorders* **20**, 353–367, 10.1177/1087054712473179 (2016). 23382578 10.1177/1087054712473179

[57] Dshemuchadse, M., Scherbaum, S. & Goschke, T. How decisions emerge: action dynamics in intertemporal decision making. *Journal of Experimental Psychology: General* **142**, 93, 10.1037/a0028499 (2013). 22612767 10.1037/a0028499

[58] Escobar, G. G., Morales-Chainé, S., Haynes, J. M., Santoyo, C. & Mitchell, S. H. Moderate stability among delay, probability, and effort discounting in humans. *The Psychological Record* **73**, 149–162, 10.1007/s40732-023-00537-1 (2023). 10.1007/s40732-023-00537-1 PMC9931166 36820275

[59] Faulkner, P., Selvaraj, S., Pine, A., Howes, O. D. & Roiser, J. P. The relationship between reward and punishment processing and the 5-HT 1 A receptor as shown by PET. *Psychopharmacology* **231**, 2579–2586, 10.1007/s00213-013-3426-9 (2014). 24429872 10.1007/s00213-013-3426-9 PMC4057624

[60] Fletcher, D., Spence, A. & Houghton, R. Thinking about your future self: Do better perspective-takers make more patient decisions? *Personality and Individual Differences* **212**, 112281, 10.1016/j.paid.2023.112281 (2023).

[61] Gassen, J. *et al*. Experimentally-induced inflammation predicts present focus. *Adaptive Human Behavior and Physiology* **5**, 148–163, 10.1007/s40750-019-00110-7 (2019).

[62] Gluth, S., Hotaling, J. M. & Rieskamp, J. The attraction effect modulates reward prediction errors and intertemporal choices. *Journal of Neuroscience* **37**, 371–382, 10.1523/JNEUROSCI.2532-16.2016 (2017). 28077716 10.1523/JNEUROSCI.2532-16.2016 PMC6596571

[63] Hare, T. A., Hakimi, S. & Rangel, A. Activity in dlPFC and its effective connectivity to vmPFC are associated with temporal discounting. *Frontiers in neuroscience* **8**, 50, 10.3389/fnins.2014.00050 (2014). 24672421 10.3389/fnins.2014.00050 PMC3957025

[64] Hartmann, M., Martarelli, C. S., Reber, T. P. & Rothen, N. Does a smartphone on the desk drain our brain? No evidence of cognitive costs due to smartphone presence in a short-term and prospective memory task. *Consciousness and cognition* **86**, 103033, 10.1016/j.concog.2020.103033 (2020). 33137560 10.1016/j.concog.2020.103033

[65] Herting, M. M., Schwartz, D., Mitchell, S. H. & Nagel, B. J. Delay discounting behavior and white matter mi-crostructure abnormalities in youth with a family history of alcoholism. *Alcoholism: Clinical and Experimental Research* **34**, 1590–1602, 10.1111/j.1530-0277.2010.01244.x (2010). 20586754 10.1111/j.1530-0277.2010.01244.x PMC3206791

[66] Jiang, T. & Dai, J. Cognitive load enhances patience rather than impulsivity. *Psychonomic Bulletin & Review* **31**, 1216–1232, 10.3758/s13423-023-02403-1 (2024). 37932578 10.3758/s13423-023-02403-1

[67] Jones, S. A., Steele, J. S. & Nagel, B. J. Binge drinking and family history of alcoholism are associated with an altered developmental trajectory of impulsive choice across adolescence. *Addiction* **112**, 1184–1192, 10.1111/add.13823 (2017). 28317212 10.1111/add.13823 PMC5461183

[68] Keidel, K., Ettinger, U., Murawski, C. & Polner, B. The network structure of impulsive personality and temporal discounting. *Journal of research in personality* **96**, 104166, 10.1016/j.jrp.2021.104166 (2022).

[69] Keidel, K. *et al*. The date/delay effect in intertemporal choice: A combined fMRI and eye-tracking study. *Human brain mapping* **45**, e26585, 10.1002/hbm.26585 (2024). 38401135 10.1002/hbm.26585 PMC10893971

[70] Knauth, K. & Peters, J. Trial-wise exposure to visual emotional cues increases physiological arousal but not temporal discounting. *Psychophysiology* **59**, e13996, 10.1111/psyp.13996 (2022). 35037293 10.1111/psyp.13996

[71] Konstantinidis, E., van Ravenzwaaij, D., Güney, S. & Newell, B. R. Now for sure or later with a risk? Modeling risky intertemporal choice as accumulated preference. *Decision* **7**, 91, 10.1037/dec0000103 (2020).

[72] Kräplin, A. *et al*. Dysfunctional decision-making in pathological gambling: pattern specificity and the role of impulsivity. *Psychiatry research* **215**, 675–682, 10.1016/j.psychres.2013.12.041 (2014). 24434041 10.1016/j.psychres.2013.12.041

[73] Kräplin, A., Scherbaum, S., Bühringer, G., Goschke, T. & Schmidt, A. Negative interpersonal scenes decrease inhibitory control in healthy individuals but not in gambling disorder patients. *International Gambling Studies*, 1–17. 10.1080/14459795.2018.1448426 (June 2018).

[74] Kräplin, A., Scherbaum, S., Bühringer, G. & Goschke, T. Decision-making and inhibitory control after smoking-related priming in nicotine dependent smokers and never-smokers. *Addictive behaviors* **88**, 114–121, 10.1016/j.addbeh.2018.08.020 (2019). 30176499 10.1016/j.addbeh.2018.08.020

[75] Kräplin, A. *et al*. The role of inhibitory control and decision-making in the course of Internet gaming disorder. *Journal of behavioral addictions* **9**, 990–1001, 10.1016/j.addbeh.2018.08.020 (2020). 33136066 10.1556/2006.2020.00076 PMC8969738

[76] Kräplin, A. *et al*. Impulsive decision-making predicts the course of substance-related and addictive disorders. *Psychopharmacology* **237**, 2709–2724, 10.1007/s00213-020-05567-z (2020). 32500211 10.1007/s00213-020-05567-z PMC7501099

[77] Lange, F. & Eggert, F. Sweet delusion. Glucose drinks fail to counteract ego depletion. *Appetite* **75**, 54–63, 10.1016/j.appet.2013.12.020 (2014). 24389240 10.1016/j.appet.2013.12.020

[78] Lempert, K. M., Glimcher, P. W. & Phelps, E. A. Emotional arousal and discount rate in intertemporal choice are reference dependent. *Journal of Experimental Psychology: General* **144**, 366–373, 10.1037/xge0000047 (2015). 25602754 10.1037/xge0000047 PMC4388786

[79] Lempert, K. M., Speer, M. E., Delgado, M. R. & Phelps, E. A. Positive autobiographical memory retrieval reduces temporal discounting. *Social cognitive and affective neuroscience* **12**, 1584–1593, 10.1093/scan/nsx086 (2017). 28655195 10.1093/scan/nsx086 PMC5647796

[80] Lempert, K. M., Lackovic, S. F., Tobe, R. H., Glimcher, P. W. & Phelps, E. A. Propranolol reduces reference-dependence in intertemporal choice. *Social Cognitive and Affective Neuroscience* **12**, 1394–1401, 10.1093/scan/nsx081 (2017). 28992268 10.1093/scan/nsx081 PMC5737445

[81] Linhartová, P. *et al*. Impulsivity in patients with borderline personality disorder: a comprehensive profile compared with healthy people and patients with ADHD. *Psychological medicine* **50**, 1829–1838, 10.1017/S0033291719001892 (2020). 31439062 10.1017/S0033291719001892

[82] Ljusic, N., Fagerstrøm, A., Sigurdsson, V. & Arntzen, E. Information, ingestion, and impulsivity: The impact of technology-enabled healthy food labels on online grocery shopping in impulsive and non-impulsive consumers. *Frontiers in Nutrition* **10**, 1129883, 10.3389/fnut.2023.1129883 (2023). 37063326 10.3389/fnut.2023.1129883 PMC10099808

[83] Lukinova, E. & Erlich, J. C. Quantifying the contribution of individual variation in timing to delay-discounting. *Scientific reports* **11**, 18354, 10.1038/s41598-021-97496-w (2021). 34526520 10.1038/s41598-021-97496-w PMC8443764

[84] Ortner, G. R. *et al*. No evidence for an effect of testosterone administration on delay discounting in male university students. *Psychoneuroendocrinology* **38**, 1814–1818, 10.1016/j.psyneuen.2012.12.014 (2013). 23339890 10.1016/j.psyneuen.2012.12.014

[85] O’Hora, D., Carey, R., Kervick, A., Crowley, D. & Dabrowski, M. Decisions in motion: Decision dynamics during intertemporal choice reflect subjective evaluation of delayed rewards. *Scientific reports* **6**, 20740, 10.1038/srep20740 (2016). 26867497 10.1038/srep20740 PMC4751609

[86] Reppert, T. R., Lempert, K. M., Glimcher, P. W. & Shadmehr, R. Modulation of saccade vigor during value-based decision making. *Journal of Neuroscience* **35**, 15369–15378, 10.1523/JNEUROSCI.2621-15.2015 (2015). 26586823 10.1523/JNEUROSCI.2621-15.2015 PMC4649007

[87] Robinson, O. J., Bond, R. L. & Roiser, J. P. The impact of threat of shock on the framing effect and temporal discounting: executive functions unperturbed by acute stress? *Frontiers in Psychology* **6**, 1315, 10.3389/fpsyg.2015.01315 (2015). 26441705 10.3389/fpsyg.2015.01315 PMC4562307

[88] Rodriguez-Moreno, D. V. *et al*. Delay discounting and neurocognitive correlates among inner city adolescents with and without family history of substance use disorder. *Developmental cognitive neuroscience* **48**, 100942, 10.1016/j.dcn.2021.100942 (2021). 33751954 10.1016/j.dcn.2021.100942 PMC8010627

[89] Scherbaum, S., Dshemuchadse, M. & Goschke, T. Building a bridge into the future: dynamic connectionist modeling as an integrative tool for research on intertemporal choice. *Frontiers in psychology* **3**, 514, 10.3389/fpsyg.2012.00514 (2012). 23181048 10.3389/fpsyg.2012.00514 PMC3502038

[90] Scherbaum, S., Frisch, S. & Dshemuchadse, M. A bird in the hand isn’t good for long. *Experimental Psychology.*10.1027/1618-3169/a000385 (2018). 29415644 10.1027/1618-3169/a000385

[91] Scherbaum, S., Frisch, S. & Dshemuchadse, M. Step by step: Harvesting the dynamics of delay discounting decisions. *Quarterly Journal of Experimental Psychology* **71**, 949–964, 10.1080/17470218.2017.1307863 (2018). 10.1080/17470218.2017.1307863 28300478

[92] Scherbaum, S., Frisch, S., Holfert, A.-M., O’Hora, D. & Dshemuchadse, M. No evidence for common processes of cognitive control and self-control. *Acta Psychologica* **182**, 194–199, 10.1016/j.actpsy.2017.11.018 (2018). 29202280 10.1016/j.actpsy.2017.11.018

[93] Schüller, C. B. *et al*. Temporal discounting in adolescents and adults with Tourette syndrome. *PLoS One* **16**, e0253620, 10.1371/journal.pone.0253620 (2021). 34143854 10.1371/journal.pone.0253620 PMC8213148

[94] Schwenke, D., Wehner, P. & Scherbaum, S. Effects of individual and dyadic decision-making and normative reference on delay discounting decisions. *Cognitive Research: Principles and Implications* **7**, 71, 10.1186/s41235-022-00422-5 (2022). 35900639 10.1186/s41235-022-00422-5 PMC9334506

[95] Soutschek, A. *et al*. Dopaminergic D1 receptor stimulation affects effort and risk preferences. *Biological Psychiatry* **87**, 678–685, 10.1016/j.biopsych.2019.09.002 (2020). 31668477 10.1016/j.biopsych.2019.09.002

[96] Soutschek, A., Moisa, M., Ruff, C. C. & Tobler, P. N. The right temporoparietal junction enables delay of gratification by allowing decision makers to focus on future events. *PLoS biology* **18**, e3000800, 10.1371/journal.pbio.3000800 (2020). 32776945 10.1371/journal.pbio.3000800 PMC7447039

[97] Soutschek, A. & Tobler, P. N. Know your weaknesses: Sophisticated impulsiveness motivates voluntary self-restrictions. *Journal of experimental psychology: learning, memory, and cognition* **46**, 1611, 10.1037/xlm0000833 (2020). 32134317 10.1037/xlm0000833

[98] Soutschek, A., Moisa, M., Ruff, C. C. & Tobler, P. N. Frontopolar theta oscillations link metacognition with prospective decision making. *Nature Communications* **12**, 3943, 10.1038/s41467-021-24197-3 (2021). 34168135 10.1038/s41467-021-24197-3 PMC8225860

[99] Soutschek, A., Bulley, A. & Wittekind, C. E. Metacognitive deficits are associated with lower sensitivity to preference reversals in nicotine dependence. *Scientific Reports* **12**, 19787, 10.1038/s41598-022-24332-0 (2022). 36396945 10.1038/s41598-022-24332-0 PMC9671892

[100] Thome, J. *et al*. Model-based experimental manipulation of probabilistic behavior in interpretable behavioral latent variable models. *Frontiers in Neuroscience* **16**, 1077735, 10.3389/fnins.2022.1077735 (2023). 36699538 10.3389/fnins.2022.1077735 PMC9868576

[101] Thrailkill, E. A., DeSarno, M. & Higgins, S. T. Loss aversion and risk for cigarette smoking and other substance use. *Drug and alcohol dependence* **232**, 109307, 10.1016/j.drugalcdep.2022.109307 (2022). 35093680 10.1016/j.drugalcdep.2022.109307 PMC8887823

[102] Thrailkill, E. A., DeSarno, M. & Higgins, S. T. Intersections between environmental reward availability, loss aversion, and delay discounting as potential risk factors for cigarette smoking and other substance use. *Preventive medicine* **165**, 107270, 10.1016/j.ypmed.2022.107270 (2022). 36152818 10.1016/j.ypmed.2022.107270 PMC10876085

[103] Thrailkill, E. A., DeSarno, M. & Higgins, S. T. Loss aversion and current, former, and never-smoking status. *Nicotine and Tobacco Research* **25**, 1277–1282, 10.1093/ntr/ntad043 (2023). 36934337 10.1093/ntr/ntad043 PMC10256887

[104] Wagner, B. J. *et al*. Chronic deep brain stimulation of the human nucleus accumbens region disrupts the stability of intertemporal preferences. *Journal of Neuroscience* **43**, 7175–7185, 10.1523/JNEUROSCI.0138-23.2023 (2023). 37684029 10.1523/JNEUROSCI.0138-23.2023 PMC10601365

[105] De Water, E. *et al*. Neural mechanisms of individual differences in temporal discounting of monetary and primary rewards in adolescents. *NeuroImage* **153**, 198–210, 10.1016/j.neuroimage.2017.04.013 (2017). 28411154 10.1016/j.neuroimage.2017.04.013

[106] Weidacker, K., Johnston, S. J., Mullins, P. G., Boy, F. & Dymond, S. Impulsive decision-making and gambling severity: The influence of *γ*-amino-butyric acid (GABA) and glutamate-glutamine (Glx). *European Neuropsychopharmacology* **32**, 36–46, 10.1016/j.euroneuro.2019.12.110 (2020). 31901336 10.1016/j.euroneuro.2019.12.110

[107] Yang, X.-L., Chen, S.-T. & Liu, H.-Z. The effect of incentives on intertemporal choice: Choice, confidence, and eye movements. *Frontiers in Psychology* **13**, 989511, 10.3389/fpsyg.2022.989511 (2022). 36405167 10.3389/fpsyg.2022.989511 PMC9671521

[108] Zgonnikov, A., Atiya, N. A., O’hora, D., Rañò, I. & Wong-Lin, K. Beyond reach: Do symmetric changes in motor costs affect decision making? A registered report. *Judgment and Decision Making* **14**, 455–469, 10.1017/S1930297500006136 (2019).

[109] Zhang, Y.-Y., Zhou, L., Li, S. & Liang, Z.-Y. Computation of subjective value does not always elicit alternative-based information searching in intertemporal choice. *Journal of Behavioral Decision Making* **35**, e2274, 10.1002/bdm.2274 (2022).

[110] Zhao, W. J., Diederich, A., Trueblood, J. S. & Bhatia, S. Automatic biases in intertemporal choice. *Psychonomic Bulletin & Review* **26**, 661–668, 10.3758/s13423-019-01579-9 (2019). 30838528 10.3758/s13423-019-01579-9

[111] Zhou, L. *et al*. “Carpe diem?”: disjunction effect of incidental affect on intertemporal choice. *Frontiers in Psychology* **12**, 782472, 10.3389/fpsyg.2021.782472 (2021). 34956000 10.3389/fpsyg.2021.782472 PMC8702439

[112] Zhou, Y.-B., Li, Q., Li, Q.-Y. & Liu, H.-Z. Evaluation scale or output format: the attentional mechanism underpinning time preference reversal. *Frontiers in Psychology* **13**, 865598, 10.3389/fpsyg.2022.865598 (2022). 35496199 10.3389/fpsyg.2022.865598 PMC9046692

[113] Amasino, D. *Amount and time exert independent influences on intertemporal choice*. 10.17605/OSF.IO/2P3DJ (Open Science Framework, 2019). 10.1038/s41562-019-0537-2 PMC8020819 30971787

[114] Calluso, C. *Interindividual variability in functional connectivity as long-term correlate of temporal discounting* version 1.0. 10.6084/m9.figshare.1246203 (Figshare, 2015). 10.1371/journal.pone.0119710 PMC4361316 25774886

[115] Calluso, C. *et al*. *Cognitive dynamics of intertemporal choice in gambling disorder version 1.0*. 10.17632/D2G69JD2PB.1 (Mendeley Data, 2020). 10.1016/j.addbeh.2020.106463 32454227

[116] Fusco Gabriele Scandola, M., Hause L, Inzlicht, M. & Aglioti, S. M. *Modulating preferences during intertemporal choices through exogenous midfrontal theta transcranial alternating current - data and analyses repository*. 10.17605/OSF.IO/5T6VZ (Open Science Framework, 2023).

[117] Sangil L & Kable, J. *Cognitive Training*. 10.18112/openneuro.ds002843.v1.0.0 (OpenNeuro, 2020).

[118] Schoemann, M., Lüken, M., Grage, T., Kieslich, P. J. & Scherbaum, S. *Validating mouse-tracking: Influence of mouse-tracking design factors on the action dynamics in intertemporal decision making*. 10.17605/OSF.IO/3W6CR (OSF, 2018).

[119] Stevens, J. R. *Data from: Intertemporal similarity: discounting as a last resort*. 10.5061/dryad.qv0sk (Dryad, 2016).

[120] Zilker, V. & Pachur, T. *Does Option Complexity Contribute to the Framing Effect, Loss Aversion, and Delay Discounting in Younger and Older Adults?*10.17605/OSF.IO/BJ2TV (Open Science Framework, 2020).

[121] Pongratz, H. & Schoemann, M. *A large-scale dataset of choice and response-time data in intertemporal choice* version 1.0. 10.17605/OSF.IO/3WSAE (Open Science Framework, 2025). 10.1038/s41597-026-06947-4 PMC12960822 41775717

[122] Pongratz, H. & Schoemann, M. *ITC Database: Choice-RT data in ITC version 1.0*. 10.17605/OSF.IO/MXUCN (Open Science Framework, 2025).

